# Wood/Dynamic Covalent Polymer Network Composites

**DOI:** 10.3390/polym18111324

**Published:** 2026-05-27

**Authors:** Jiaxi Kuang, Wanting Wang, Shuqi Shang, Ziyi Yan, Lianpeng Zhang, Kaimeng Xu, Linkun Xie, Huanbo Wang, Tian Liu

**Affiliations:** 1 Yunnan Provincial Key Laboratory of Wood Adhesives and Glued Products, International Joint Research Center for Biomass Materials, Southwest Forestry University, Kunming 650224, China; 15179531061@163.com (J.K.);; 2 Key Laboratory of Bio-Based Material Science and Technology, Engineering Research Center of Advanced Wooden Materials, Ministry of Education, Northeast Forestry University, Harbin 150040, China

**Keywords:** wood, dynamic covalent polymer networks, composites, shape-memory, reprocessibility

## Abstract

Wood, a renewable and sustainable resource with a hierarchical porous structure, exhibits significant potential for functional composites through integration with polymers. Wood/polymer composites are typically fabricated either via polymer impregnation into wood or through blending of wood powder with thermoplastic polymers to produce wood–plastic composites (WPCs). However, conventional thermosetting polymers cannot be reshaped or reprocessed, while thermoplastic polyolefins often exhibit poor compatibility with wood powder. Dynamic covalent polymer networks (DCPNs), which incorporate reversible covalent bonds into thermoset matrices, enable network reconfiguration in response to external stimuli such as heat. Replacing conventional polymers with DCPNs in delignified wood yields transparent wood with programmable shape-memory, photo-luminescent, and thermochromic properties, enabling the fabrication of advanced materials. DCPN-impregnated delignified wood is also reprocessable and degradable. Similarly, incorporating DCPNs into carbonized wood produces electrode materials with enhanced plasticity, shape-memory behavior, reshaping ability, and self-healing properties. DCPNs can replace thermoplastic polyolefins as matrices in WPCs. Consequently, repairable and reprocessable wood powder/DCPN composites can be fabricated with potential for carbon storage applications. This mini-review summarizes recent advances in wood/DCPN composites, focusing on two main fabrication approaches: DCPN impregnation into delignified wood and blending of DCPNs with wood powder. Wood/DCPN composites combine the characteristics of wood and dynamic DCPNs and have the potential to become an efficient, eco-friendly, and sustainable form of processing and utilization of wood.

## 1. Introduction

Wood is a natural, renewable composite material with a high strength-to-weight ratio, excellent elasticity, strong impact resistance, aesthetic grain patterns, and biodegradability. At the molecular scale, wood mainly consists of intertwined macromolecules formed by cellulose and hemicellulose polysaccharides, along with amorphous aromatic lignin. At the cellular scale, wood exhibits a porous structure composed of an extensive network of interconnected cells [1,2]. The hierarchical structure and chemical composition of wood impart high strength and stiffness at low density [3]. Wood serves as a natural carbon reservoir, with carbon accounting for up to 50% of its composition. Wood sequesters carbon through the conversion of atmospheric CO_2_ into sugars, oxygen, and organic compounds via photosynthesis [4,5]. Therefore, maximizing wood utilization is crucial for reducing reliance on fossil fuels and mitigating carbon emissions [6].

Wood is prone to deformation and cracking under fluctuating humidity owing to its hydrophilicity. Moreover, wood, a predominant organic material, is susceptible to attack by microorganisms and termites. Fast-growing timber often exhibits low density, poor strength, low hardness, and a tendency to fibrillate during processing owing to its short growth period. Chemical impregnation enhances wood quality through the modification of cell walls and filling of voids. The impregnation process typically employs vacuum, atmospheric pressure, compression, or their combinations to introduce chemical agents into the wood [7]. This process alters or expands the cell wall structure, thereby enhancing overall wood performance and durability. Chemical impregnation can enhance the dimensional stability, mechanical properties, flame retardancy, and resistance of wood to decay and insect damage. Thermosetting resins (phenolic, urea-formaldehyde, and melamine-formaldehyde) are commonly used for wood impregnation. Low-molecular-weight resins or their monomers are introduced into wood via vacuum–pressure impregnation and then cured via heating, microwave irradiation, or thermal radiation. This process forms water-insoluble, crosslinked polymers that expand the cell walls and improve dimensional stability. Impregnation of thin wood veneers with low-molecular- weight phenolic resins and sub-sequent multilayer assembling and hot pressing produces materials with ultrahigh hard-ness and strong puncture resistance [8].

Additionally, similar to most organic network materials, wood undergoes gradual decomposition and carbonization upon heating. Unlike metals, glasses, or thermoplastics, wood cannot be processed or recycled using thermoplastic methods. Conventional wood processing mainly relies on sawing, planning, and sanding, which result in material loss, particularly at smaller scales. Overall, production losses can exceed 50% of the original timber volume. The conversion of wood into thermoplastic materials enables continuous processing such as extrusion, hot pressing, and injection molding, thereby enhancing resource efficiency. Moreover, these materials can be recycled through thermoplastic processes, thereby im-proving the sustainable, low-carbon utilization of wood and supporting a circular economy [4]. Wood–plastic composites (WPCs), produced from biomass materials (e.g., wood flour) and thermoplastic polymers via melt blending, enable thermoplastic processing and recycling. These composites provide an environmentally friendly alternative that combines the ad-vantages of biomass and thermoplastics. The biomass component in WPCs may include wood-processing by-products, agricultural straw, or other natural fibers. The thermoplastic matrix in WPCs typically consists of widely used polymers, including polyethylene, polypropylene, polyvinyl chloride, and polystyrene. WPCs are widely used in transportation, outdoor decking, automotive interiors, indoor flooring and decoration, landscaping, and fencing.

Biomass materials contain abundant hydroxyl groups and exhibit strong polarity, leading to poor compatibility with generally nonpolar polyolefin-based polymers [9]. This incompatibility adversely affects the mechanical and hygroscopic properties of the resulting composites. Although interfacial compatibilizers are added during WPC processing, they only partially improve adhesion between wood flour and the polymer matrix. At high wood flour contents, the polymer matrix becomes insufficient to form a continuous phase, which exacerbates interfacial issues and limits WPC performance. Moreover, higher wood flour content increases melt viscosity and reduces flowability, which hinders material processability. Consequently, the wood flour content in polyolefin-based composites is typically limited to below 60% in industrial applications. Because the polyolefin matrix is mechanically inferior to wood, WPCs exhibit reduced overall mechanical performance [10,11].

Therefore, developing high-performance, reprocessable polymers compatible with polar biomass is crucial for fabricating advanced wood-based composites. This approach can enhance wood value, reduce resource waste, and mitigate environmental impacts. Dynamic covalent polymer networks (DCPNs), with reversible bonds, can be reshaped and recycled, making them promising candidates. The polar nature of DCPNs ensures good compatibility with biomass materials. Accordingly, this mini-review focuses on wood–DCPN composites, particularly DCPN-impregnated wood and wood powder/DCPN composites.

## 2. Dynamic Covalent Polymer Networks

In recent decades, dynamic chemical bonds (including supramolecular interactions and reversible covalent bonds) have been incorporated into polymer networks to overcome the limitations of conventional polymers. These modifications enable polymers to exhibit macroscopic responses to external stimuli, including heat, light, electricity, moisture, magnetic fields, and pH changes [12,13,14]. Polymers containing these dynamic bonds are referred to as “dynamic polymers” [13] or “adaptable networks” [15,16]. DCPNs are dynamic polymers based on reversible covalent bonds [17,18].

### 2.1. Conventional Polymers

Polymers are typically classified as thermoplastics and thermosets based on their molecular structure and rheological behavior. Thermoplastics consist of linear, branched, or dendritic chains that can flow upon heating. Consequently, thermoplastics can be processed and shaped via extrusion, injection molding, and compression molding. Additionally, thermoplastic waste can be recycled and reused. In contrast, thermosets form three-dimensional, crosslinked network structures and are typically produced via curing of liquid resins with hardeners under controlled conditions. Once cured, thermosets cannot be reprocessed or remolded via reheating. The properties of thermoplastics depend on their chemical structure and molecular weight. Achieving high molecular weight in thermoplastics requires harsh synthesis conditions, such as high temperatures and pressures, with precise control of raw material ratios. Consequently, polymer synthesis and processing are conducted as separate steps. Furthermore, high-molecular-weight thermoplastics exhibit high melt viscosity, which significantly changes near the glass transition temperature (*T_g_*). This results in a narrow processing temperature window and poor melt wettability. In contrast, thermosets are synthesized from low-viscosity multifunctional monomers at room temperature or under heating to form crosslinked networks with effectively infinite molecular weight. These monomers exhibit strong polarity, low viscosity, and excellent wettability toward fibrous materials. Consequently, high-performance composites (e.g., glass fiber, carbon fiber, and hemp fi-ber composites) are predominantly based on thermosets. However, despite their widespread use in aerospace, wind turbine blades, and automo-tive interiors, these composites cannot be repaired once damaged.

### 2.2. Dynamic Covalent Polymer Networks with Dissociative Exchange Mechanisms

**Figure 1:**
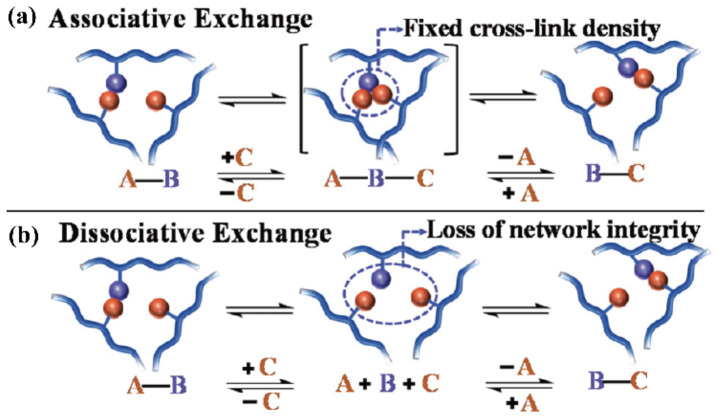
(**a**) Associative and (**b**) Dissociative exchange mechanisms in DCPNs. Reproduced with permission [20]. Copyright 2022, Royal Society of Chemistry.

DCPNs with exchangeable covalent bonds can be classified into two types based on their bond-exchange mechanisms: dissociative and associative exchange. Polymers containing these bonds are referred to as dissociative and associative dynamic polymers (Figure 1) [15,19,20]. The reversible bonds of associative DCPNs cleave and reform in a single step, maintaining the integrity of their networks and keeping the viscosity of the DCPNs constant. In contrast, the reversible bonds of dissociative DCPNs cleave and reform step by step, leading to the destruction of their network integrity and a sharp drop in the viscosity of the DCPNs. For example, dissociative DCPNs contain conjugated dienes and monomers capable of undergoing reversible Diels–Alder (D–A) reactions [15,19]. Upon heating, the equilibrium shifts toward the retro D–A reaction, leading to bond cleavage. The accelerated breaking and reformation of polymer chains promote network dissociation. As network continuity decreases, the material structure becomes disordered, leading to stress relaxation and increased flowability. This temporary reduction in crosslink density causes a sharp decrease in viscosity at high temperatures, with behavior similar to that of thermoplastics. Upon cooling, the equilibrium shifts back toward the D–A addition reaction. The crosslinked network reforms with a degree of crosslinking comparable to that of the original material. This heating-induced dissociation and cooling-induced re-crosslinking behavior enable repeated processing and reshaping of the polymer network.

**Figure 2:**
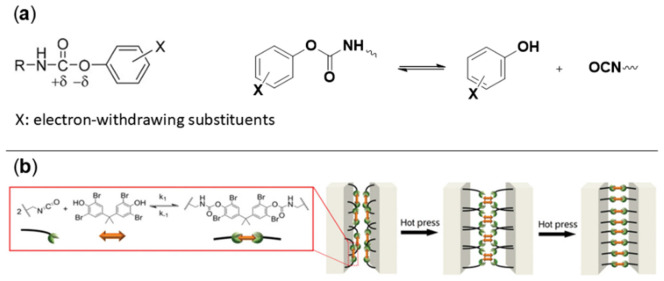
(**a**) Effects of electron-withdrawing groups on phenol–carbamate bond dissociation. (**b**) Schematic of hot pressing of DCPNs containing electron-withdrawing groups. Reproduced with permission [43]. Copyright 2020, Wiley-VCH.

Similarly, various dynamic chemical bonds have been incorporated into dissociative exchange networks, including triazolinediones [21], hindered urea bonds [22], reversible C–C bonds [23], alkoxyamine bonds [24,25], guanidine C-N bonds [26], C=N iminoboronate bonds [27,28], oxime–carbamate bonds [29,30], anilinium salts [31], hydroxyl acetals [32,33], primary amine acetals [34], and ortho-benzoate esters [35,36,37]. DCPNs containing reversible phenol–carbamate bonds, formed via reactions between phenolic hydroxyl groups and isocyanates, have attracted significant research interest [38,39,40,41]. Phenol–carbamate bonds were first used as “capping agents” in polyurethane synthesis to suppress side reactions with isocyanates and control reaction progression [42]. Phenol–carbamate bonds were first used as “capping agents” in polyurethane synthesis to suppress side reactions with isocyanates and control reaction progression. Modifying the type and position of these substituents regulates phenol–carbamate bond dissociation, enabling precise control over the properties of the resulting dynamic polymers. For example, electron-withdrawing groups reduce the dissociation temperature of phenol–carbamate bonds. Conversely, electron-donating groups increase the dissociation temperature [40]. Electron-withdrawing substituents reduce charge separation between the carbonyl carbon and the oxygen atom, thereby weakening the phenol–carbamate bonds (Figure 2a). Moreover, the incorporation of tertiary amine catalysts enhances the efficiency of bond cleavage and reformation [43]. Consequently, DCPNs containing phenol–carbamate bonds can be remolded via hot pressing at moderate temperatures (110 °C) (Figure 2b). Natural phenolic compounds derived from biomass, such as tannic acid [44], propyl gallate [45,46], and gallic acid [40], have strong potential for the synthesis of phenol–carbamate-based DCPNs. These findings highlight the potential of these DCPNs in bio-based dynamic polymer systems.

### 2.3. Dynamic Covalent Polymer Networks with Associative Exchange Mechanisms

In associative DCPNs, heating does not cause depolymerization, and both the cross-link density and network integrity are maintained (Figure 1b). In these systems, bond breaking and formation occur simultaneously at the same site, thereby preserving the structural stability of DCPNs. Early associative DCPNs include allyl sulfide-derived systems formed via light- or initiator-induced radical addition and segment transfer [47,48]. These materials exhibit stress relaxation, flowability, and self-healing. However, the performance of DCPNs can be limited by radical termination reactions.

**Figure 3:**
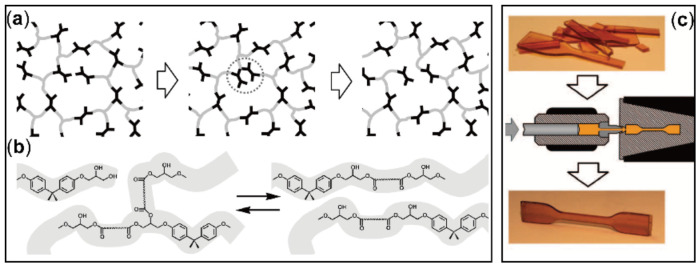
(**a**) Topological rearrangement of vitrimer networks at high temperatures. (**b**) Transesterification in vitrimers. (**c**) Remolding of vitrimers. Reproduced with permission [51]. Copyright 2011, The American Association for the Advancement of Science.

In 2011, Ludwik Leibler incorporated an ester exchange catalyst, zinc acetylacetonate, into epoxy/carboxylic acid and epoxy/anhydride curing systems to create temperature-responsive associative DCPNs (Figure 3) [46]. Owing to their SiO_2_-like viscosity behavior near the glass transition temperature, distinct from those of thermoplastics and thermosets, these materials were termed “vitrimers,” or glass-like polymers [49]. Vitrimers are covalent polymer networks that undergo topological rearrangement via thermally activated associative exchange and exhibit thermoplastic behavior [19]. As temperature increases, the viscosity of vitrimers decreases according to the Arrhenius equation. This indicates that the rheological properties of vitrimers are mainly controlled by associ-ative exchange reactions. In contrast, conventional thermoplastics and dissociative DCPNs undergo a rapid solid–liquid transition. The viscosity–temperature relationship of conventional thermoplastics and dissociative DCPNs are described by the Williams–Landel–Ferry equation. Additionally, vitrimers exhibit two temperature-dependent transitions. The glass transition temperature (*T_g_*) corresponds to the onset of segmental motion, while the topological transition temperature (Tv) is associated with bond-exchange reactions [50]. As bond exchange occurs faster than deformation, the network topology becomes reconfigurable, enabling material flow. *T_v_* is defined as the temperature at which the viscosity reaches 10^12^ Pa·s [50,51]. Because vitrimers maintain a constant crosslink density, they swell but do not dissolve in solvents, regardless of temperature [51,52]. Therefore, the chemical reactions, segment mobility, and rheological behavior of vitrimers significantly differ from those of conventional thermoplastics, thermosets, and dissociative DCPNs. In addition to ester exchange, vitrimer systems have been developed based on alkyl exchange reactions (e.g., triazole salts) [53], amino exchange reactions in vinyl polyurethanes [54], and dioxaborolane exchange reactions [52]. Besides, materials akin to vitrimers have also been reported [55,56,57]. However, epoxy-based vitrimers relying on ester exchange remain the most widely used [58,59,60].

## 3. Dynamic Covalent Polymer Network-Infiltrated Wood Composites

**Figure 4:**
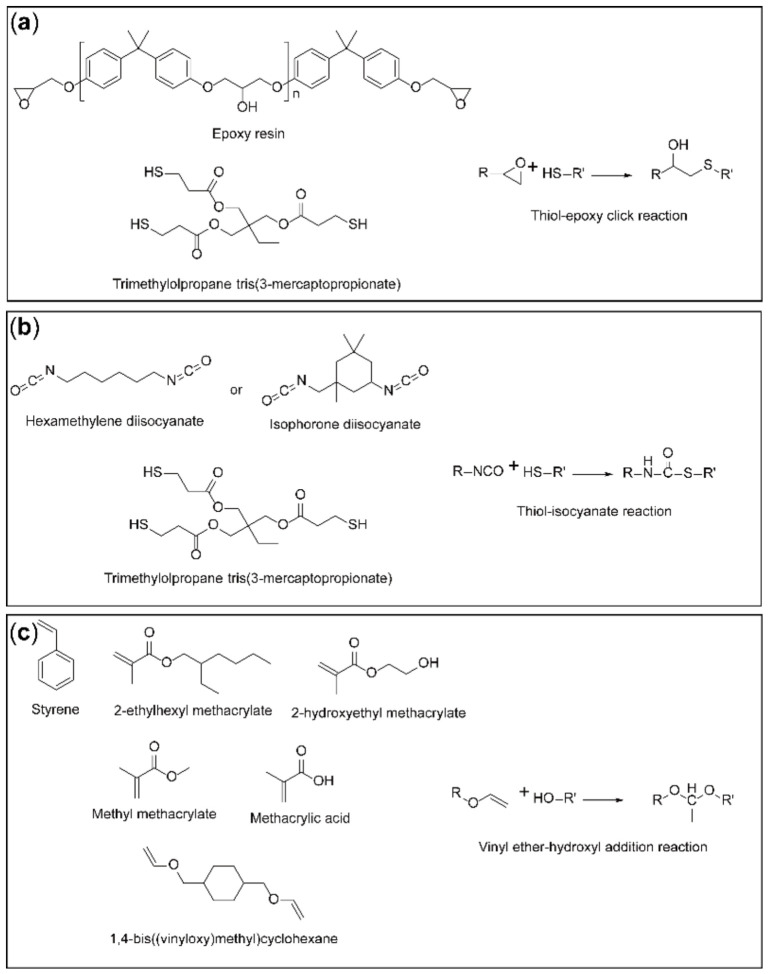
Monomers and reaction types in DCPNs-infiltrated wood composites: (**a**) epoxy resin and trisulfide via thiol-epoxy click reaction; (**b**) isocyanate and trisulfide via thiol-isocyanate reaction; (**c**) vinyl monomer via vinyl ether-hydroxyl addition reaction.

Wood is a porous material with multiscale pore structures. Impregnating polymers into these pores enhances dimensional stability and imparts unique functionalities. Removal or modification of lignin and impregnation with refractive index-matched pol-ymers enable the fabrication of transparent wood (TW) [61]. Replacing conventional polymers with DCPNs in delignified wood yields TW with added functionalities, such as shape- memory, photoluminescent, and thermochromic properties [62,63,64]. Common monomers used to construct DCPNs include epoxy resins, isocyanates, and vinyl monomers. These monomers are crosslinked with trimethylolpropane tris(3-mercaptopropionate) or 1,4-bis((vinyloxy)methyl)cyclohexane via thiol-epoxy click reaction, thiol-isocyanate reaction, and vinyl ether-hydroxyl addition reaction, to form DCPNs (Figure 4). These monomeric reactions occur under moderate conditions that facilitate the construction of polymer networks. The introduced dynamic ester bonds, thiocarbamate, and acetal linkages in the constructed polymer networks enable topology reconfiguration under certain stimuli.

Shape-memory materials can adopt temporary shapes and revert to their original configurations upon exposure to external stimuli such as heat, light, or magnetic fields [61,63]. DCPNs exhibit significant thermoplasticity through heat-activated topological rear-rangements. A poly-caprolactone-based network with crystalline phase transitions was synthesized via a thiol–ene click reaction, and an organic base catalyst was incorporated into the system. At high temperatures (130 °C), the catalyst induces ester exchange reactions, leading to topological rearrangement of the crosslinked network and enabling plasticity (i.e., permanent shape change). At lower temperatures (70 °C), only segmental motion is activated, and the deformation can be fixed upon cooling. Upon reheating, the segments regain mobility. The material recovers its original shape owing to entropy-driven relaxation, indicating the shape-memory effect [65]. This approach enables two distinct behaviors—temporary (elastic) and permanent (plastic) deformation—in the same material at different temperatures. Notably, the material undergoes plastic deformation but remains in the solid state. This phenomenon is referred to as solid-state plasticity. Through thermally induced plasticity, complex geometries can be achieved without molds, including hard-to-fabricate shapes. These polymers exhibit a unique shape accumulation effect. During successive plastic deformations, the previous permanent shape is not erased but incorporated into the subsequent shape. This accumulation enables the stepwise construction of increasingly complex geometries, highlighting the advantages of thermally induced plasticity.

**Figure 5:**
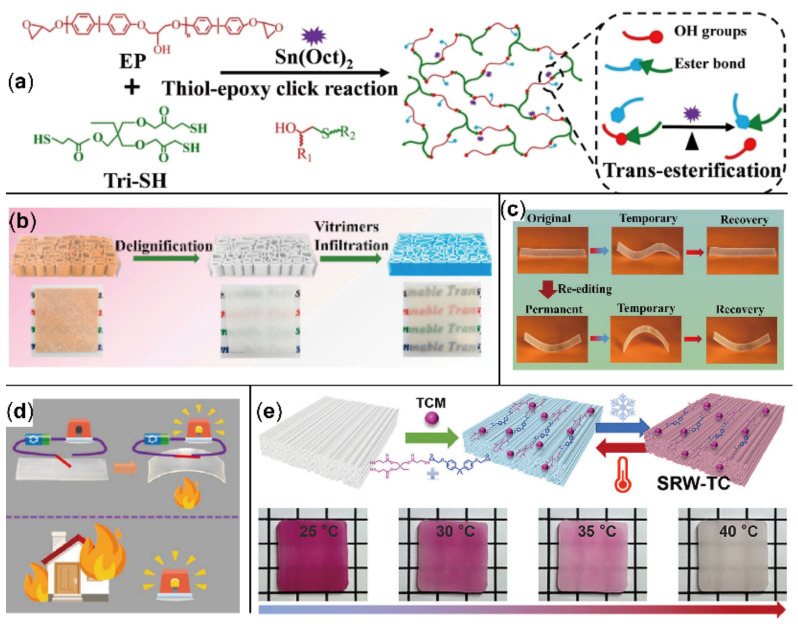
Demonstrations of shape-memory TW: (**a**) Reaction and transesterification mechanisms of epoxy vitrimers. Reproduced with permission [64]. Copyright 2024, Wiley-VCH. (**b**) Fabrication procedure of vitrimer-impregnated TW. Reproduced with permission [63]. Copyright 2021, Elsevier. (**c**) Schematic of the editable shape-memory behavior of TW. Reproduced with permission [66]. Copyright 2022, Springer Nature. (**d**) Fire-alarm actuation mechanism of TW. Reproduced with permission [67]. Copyright 2022, Elsevier. (**e**) Fabrication process and color-change demonstration of shape–color dual-responsive wood. Reproduced with permission [62]. Copyright 2024, American Chemical Society.

Wang et al. developed TW with programmable shape-memory behavior by intro-ducing transparent, refractive index-matched, epoxy-based vitrimers into delignified wood (Figure 5) [63]. When TW cools to room temperature, their shape is fixed, and shape recovery occurs upon heating. The incorporated vitrimer network imparts multiple programmable functions, including shape recovery, shape programming, shape erasing, reprogramming, and reversible transformation between temporary and permanent shapes. The resulting TW (2 mm thick) exhibits 60% transmittance and 95% haze. Wang et al. further *T_g_* from ~30 °C to 50 °C and enhanced the mechanical properties of TW by increasing the crosslink density of the vitrimer network [66]. This was achieved via substitution of a trifunctional thiol with a tetrafunctional thiol crosslinker. The resulting TW is stiff at low temperatures and flexible at high temperatures. Additionally, TW demonstrates high haze and transmittance, excellent solid-state plasticity, and shape-manipulation capability under thermal stimuli. These features enable light guiding, directional scattering, and tunable light transmission. Moreover, TW exhibits excellent thermal insulation, indicating its strong potential for building energy-efficient applications. Wang et al. developed a smart actuator based on the reconfigurable shape-memory properties of TW. This actuator provides fire warning and is fabricated via infiltration of poly-thiourethane covalent adaptable networks into delignified wood scaffolds (Figure 5d) [67]. The resulting TW demonstrated excellent reconfigurable shape-memory behavior, strong optical performance, low thermal conductivity, and adequate mechanical strength. Similarly, shape-manipulable TW can be fabricated via a solvent-free thiol–isocyanate click reaction under low-temperature solidification (up to 60 °C) [68]. Dynamic hemiacetal ester networks, formed through addition reactions between vinyl ether bonds and hydroxyl groups, serve as vitrimer matrices in shape-memory TW. This approach achieves comparable overall performance and eliminates the need for a catalyst. Additionally, incorporating BODIPY (4,4-difluoro-4-bora-3a,4a-diazas-indacene) dyes into vitrimer networks within delignified wood templates enables the fabrication of dual-functional TW composites with photoluminescent and shape-editable properties [64]. Programmable shape–color dual-responsive wood can be fabricated through the impreg-nation of thermochromic microcapsule-doped dynamic covalent vitrimers into delignified wood. This system enables the visualization of mechanical behavior and precise control of shape-memory performance under thermal stimuli (Figure 5e) [62]. These functional shape-memory TW materials may promote the development of advanced wood-based composites for applications such as material interactivity, environ-mental sensing, performance visualization, soft actuation, information encryption, and shape–color control [69,70].

**Figure 6:**
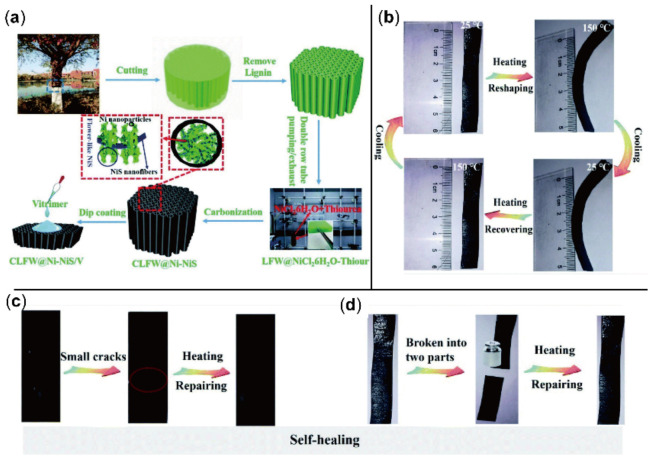
(**a**) Schematic of the fabrication process of the CLFW@Ni–NiS/V hybrid. (**b**) Shape-memory and reshaping properties at a thickness of 3 mm. Self-healing ability of the CLFW@Ni–NiS/MIV hybrid under different damage conditions: (**c**) partial cracks and (**d**) breakage into two parts. Reproduced with permission [71]. Copyright 2020, Royal Society of Chemistry.

Wood with good conductivity can be used in energy storage and catalysis. However, carbonized wood with improved conductivity is brittle and exhibits poor cycling stability in energy storage applications. To address this limitation, Xiong et al. incorporated a vitrimer into carbonized wood to construct a multifunctional composite, carbonized lignin-free wood @Ni–NiS/vitrimer(CLFW@Ni–NiS/V, Figure 6) [71]. The incorporation of vitrimers endows the electrode material with high plasticity, shape-memory behavior, reshaping ability, and self-healing, enabling adaptability to different application scenarios through deformation. The symmetric supercapacitor, assembled using the CLFW@Ni–NiS/V, exhibits high gravimetric, areal, and volumetric energy densities of 38 Wh/kg, 687 μW·h/cm^2^, and 58 W·h/L, along with high power densities of 56 kW/kg, 202 mW/cm^2^, and 39 kW/L. The strength and plasticity provided by the vitrimers prevent the electrode materials from falling off and extend the lifespan of the supercapacitors. Infiltration of delignified wood frameworks with DCPNs enables composite reprocessability via reversible exchange reactions [72]. This material exhibits interlayer damage recovery, with bending strength restored to 63.87% in damaged specimens. However, the tensile strength of the reprocessed material decreases to 17.34 MPa owing to wood fiber fracture during mechanical processing. The reprocessed material exhibits no significant decline in mechanical performance over multiple reprocessing cycles. As delignified wood is impregnated with an epoxy vitrimer via transesterification, the compo-site becomes degradable in ethylene glycol. Consequently, the epoxy vitrimer–delignified wood composite is degradable and can be thermally welded and reprocessed via hot pressing [73,74].

DCPN-infiltrated wood composites combine the metrics of the wood skeleton and dynamic DCPNs. The wood substrate form, reversible bonds of DCPNs, functionalities, and performance metrics of DCPN-infiltrated wood composites are summarized in Table 1. Self-healing, editable shape-memory, repairability, weldability, and reprocessability are unique performances of DCPN-infiltrated wood composites enabled by the introduction of DCPNs. DCPNs synthesized from low-viscosity monomers are suitable for impregnation into wood to prepare DCPN-infiltrated wood composites. DCPNs with other functionalities such as flame retardancy, bacteriostasis, antiseptic properties, energy storage, and photo-responsiveness are expected to be used to prepare multi-functional DCPN-infiltrated wood composites in future research.

## 4. Wood Powder/Dynamic Covalent Polymer Network Composites

Wood processing faces challenges owing to the lack of intensive, efficient, low-carbon, and sustainable utilization methods. Therefore, new strategies are required to improve wood processing efficiency and maximize resource utilization, particularly for underutilized materials such as poplar, small-diameter timber, irregular wood pieces, and processing residues. To ensure compatibility with these feedstocks, the size of the raw components should not exceed the smallest dimension of the wood material. According to this concept, several engineered wood products have been developed. These include reconstituted wood panels from small-diameter timber, laminated veneer lumber from wood veneers, particleboard from wood particles, and WPCs from wood powder. These materials are widely used in industry and have gained strong market acceptance. However, polyolefin-based WPCs often exhibit poor interfacial bonding, particularly at high wood powder loadings, which limits their application in high-performance structural materials. DCPNs, with thermally activated reversible bonds, exhibit self-healing, reprocessability, and degradability, making them promising matrix materials for WPCs. Moreover, these networks are based on polar molecular chains, which enhance compatibility with wood powder and facilitate the fabrication of reprocessable composites with improved performance.

**Figure 7:**
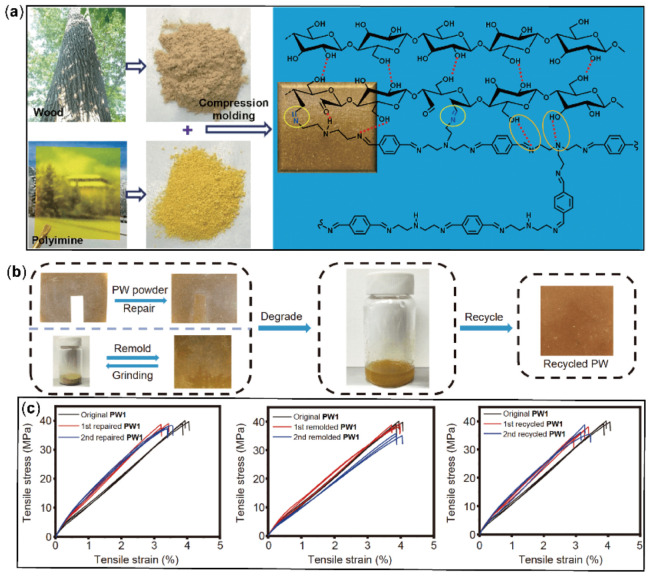
Preparation and characteristics of the wood powder/dynamic polyimine films: (**a**) Schematic representation of the preparation of the wood powder/dynamic polyimine films; (**b**) Repair, remolding, and recycling of wood powder/dynamic polyimine films; (**c**) Tensile stress-strain curves of the repaired, remolded, and recycled wood powder/dynamic polyimine films. Reproduced with permission [75]. Copyright 2020, Springer Nature.

Su et al. combined wood powder with DCPNs to fabricate composites [75]. Moreover, DCPNs were synthesized via a Schiff base reaction between terephthaldehyde and amines, including diethylenetriamine, bis(3-aminopropyl)amine, 3,3′-diamino-N-methyldipropylamine), and tris(2-aminoethyl)amine. The wood powder was oxidized with NaIO4 to introduce aldehyde groups on its surfaces, enabling participation in imine exchange reactions with the polyimine network. The resulting wood/dynamic polyimine composites were fabricated via compression molding of the oxidized wood powder with the polyimine matrix. These composites exhibited repairability, reprocessibility, and closed-loop full recyclability. The repaired, remolded, and recycled samples retained their tensile properties (Figure 7). This is mainly due to the excellent reversible ability of imine bonds and the good compatibility between wood powder and polyimine. Su et al. synthesized a cellulose-based dynamic imine polymer (Cel-DIP) matrix via a Schiff base reaction between oxidized cellulose and 1,6-hexamethylenediamine to enhance the sustainability of wood/dynamic polyimine composites [76]. The wood powder was incorporated into the Cel-DIP matrix to produce sustainable and degradable composites. The resulting wood powder/Cel-DIP composites exhibited ultrahigh tensile strength (56.1 MPa), flexural strength (94.9 MPa), Young’s modulus (2.5 GPa), and flexural modulus (10.3 GPa). These composites demonstrated excellent thermostability, water resistance, and chemical degradability. Furthermore, a fully sustainable, bio-based wood powder/polyimine vitrimer (Bio-PI) composite was developed through the integration of wood powder into a bio-derived polyimine vitrimer [77]. Bio-PI was synthesized via crosslinking of oxidized microcrystalline cellulose with a bio-based diamine (Priamine 1075). Natural, oxidized, and aminated wood powders were incorporated into Bio-PI and hot-pressed into lignocellulose-based composite films. The resulting films exhibited good flexibility and strength, with a tensile strain of 61%, toughness of 365 MJ m^−3^, and tensile strength of 9 MPa.

**Figure 8:**
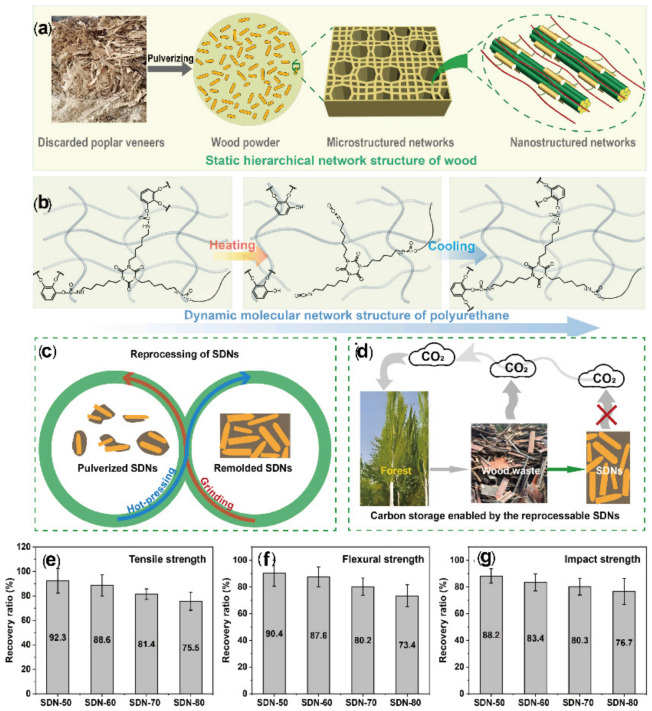
Preparation and characteristics of static–dynamic hybrid networks SDNs: (**a**) Schematic of the hierarchical wood structure with micro- and nanoscale networks; (**b**) Reversible molecular network of dynamic polyurethane during heating and cooling; (**c**) Closed-loop reprocessing process; (**d**) Schematic of carbon storage in reprocessable SDNs; (**e**) Tensile strength recovery ratio, (**f**) flexural strength recovery ratio, and (**g**) impact strength recovery ratio of reprocessed SDNs. Reproduced with permission [79]. Copyright 2025, Elsevier.

Zhao et al. fabricated wood powder/DCPN composites with high mechanical performance using an epoxy vitrimer matrix. The effects of the epoxy-to-anhydride molar ratio, hot-pressing conditions, and cyclic re-processing on the mechanical properties of the composites were systematically investigated [78]. The optimal epoxy-to-anhydride molar ratio was 1:1, with hot pressing conducted at 150 °C under 12 MPa for 30 min. Under these conditions, the composite exhibited tensile strength, tensile modulus, flexural strength, and flexural modulus of 47.3 MPa, 9.3 GPa, 79.2 MPa, and 8.9 GPa, respectively. The composites also exhibited good reprocessability, retaining 67.8% and 84.2% of their flexural strength and modulus, respectively, after cyclic reprocessing. Wang et al. further designed robust, reprocessable wood/dynamic polyurethane composites with integrated integral static-dynamic hybrid networks (SDN) based on the hierarchical micro- and nanostructures of wood and the molecular characteristics of dynamic polyurethane (Figure 8) [79]. During fabrication, hot pressing induced wood powder orientation and densification in SDNs, which increased the contact area and friction between microfibrils in the cell walls. Additionally, the dynamic polyurethane formed dynamic covalent (phenol-carbamate) and hydrogen bonds. These interactions enhanced interfacial adhesion between the wood powder and the polymer matrix. Consequently, SDNs demonstrated high mechanical performance, with tensile and flexural strengths of 55.37 ± 4.49 MPa and 88.22 ± 3.67 MPa, respectively. SDNs also exhibited good reprocessability, retaining 70% of their mechanical properties after three reprocessing cycles. The reprocessable SDNs can retain carbon from wood waste, indicating their potential for carbon storage applications.

Although it is in the incipient stage of wood powder/DCPN composites, DCPNs with imine bonds, carboxylic ester, or phenol carbamate have been successfully used as a matrix to prepare composites with wood powder (Table 2). Using DCPNs as a matrix not only avoids the defects of applying polyolefins, but also allows for chemical degradation. Compared to the reversible bonds in the DCPN matrix, imine bonds and phenol carbamate become reversible at moderate temperatures without using a catalyst. Phenol carbamate and carboxylic ester can undergo exchange reactions with the hydroxy groups of wood, which eliminates the need to modify the wood, as is necessary when using imine-containing DCPNs. Except for the three reversible bonds, a great variety of reversible bonds have been applied to construct DCPNs. Thus, DCPNs with other reversible bonds are expected to be introduced as the matrix of wood powder/DCPN composites to achieve better performance metrics. Furthermore, as melt extrusion is the main processing method for WPCs available in the market, the melt extrusion of wood powder/DCPN composites should be conducted, and their rheological properties should be investigated.

## 5. Conclusions and Future Trends

Efficient and sustainable processing and utilization of wood has long been key focus areas in forest product science. This requires full utilization of wood resources, including poplar and other underutilized materials such as small-diameter timber, irregularly shaped wood, and processing residues. DCPNs, constructed via reversible covalent bonds, exhibit unique properties. The monomers of DCPNs demonstrate good flowability and wettability at room temperature. After crosslinking, these polymers can undergo plastic reprocessing via thermo-plastic-like methods such as hot pressing, extrusion, or injection molding upon heating. Additionally, DCPNs can be dissolved or degraded in suitable solvents, enabling material recovery through dynamic bond exchange. DCPNs typically contain abundant polar functional groups, which enhance compatibility with wood materials. The dynamic bond exchange reactions of DCPNs enable repeated processing and reshaping, making them promising for wood adhesion applications. However, research in this area remains limited. This mini-review summarizes recent advances in wood/dynamic covalent polymer composites, focusing on two main fabrication approaches. First, dynamic covalent polymers are impregnated into delignified wood to fabricate TW. Second, DCPNs are blended with wood powder to form composites. Replacing conventional polymers with DCPNs in delignified wood yields TW with programmable shape-memory, photoluminescent, and thermochromic properties. These features enable the development of advanced materials. This transparent wood demonstrated up to 90% transmittance and a shape recovery ratio of up to 97.8%. DCPN-impregnated delignified wood exhibits reprocessability and degradability. Incorporating DCPNs into carbonized wood further produces electrode materials with high plasticity, shape-memory behavior, reshaping ability, and self-healing properties. Moreover, DCPNs can replace conventional thermoplastic polyolefins as matrices in WPCs. Consequently, repairable and reprocessable wood powder/DCPN composites can be fabricated with potential for carbon storage applications. Recent studies have highlighted the potential of these approaches. However, the current DCPNs are mainly synthesized from petroleum-based monomers that are toxic, which limits the application of wood/DCPN composites. Sustainable DCPNs sourced from biobased materials that are non-toxic or low-toxic present promising alternatives. Future research should focus on developing novel DCPNs with specialized functionalities, advancing wood processing and modification techniques, and expanding strategies for integrating wood with dynamic covalent polymers. These directions provide strong potential for further progress in this field. Furthermore, converting the molecular network of wood to DCPNs in situ by introducing reversible chemical bonds is a more direct and concise method for preparing wood/DCPN composites, but it is still challenging.

## Figures and Tables

**Table 1:** Wood substrate form, reversible bonds of DCPN, functionalities, and performance metrics of DCPN-infiltrated wood composites.

Wood Substrate Form	Reversible Bonds of DCPN	Functionalities	Performance Metrics	Ref.
Carbonized wood	Carbamate	Self-healing ability	Mechanical properties and CV curves were mostly retained.	[71]
Delignified wood	Carboxylic ester	Transparent and shape-memory	60% transmittance and shape recovery ratio up to 97.8%.	[63]
Delignified wood	Thiocarbamate	Transparent, shape-memory, and fire alarm	90% transmittance and shape recovery ratio up to 96.7%.	[67]
Delignified wood	Carboxylic ester	Transparent, shape-memory, and thermal insulation	60% transmittance, shape recovery ratio up to 94.4%, and thermal conductivities as low as 0.2898 W/m∙K.	[66]
Delignified wood	Thiocarbamate	Transparent, shape-memory, and thermal insulation	89.92% transmittance, shape recovery ratio up to 95%, and thermal conductivities as low as 0.30 W/m∙K.	[68]
Delignified wood	Carboxylic ester	Photoluminescent	Maximum emission peak located at 553 nm	[64]
Delignified wood	Acetal linkages	Transparent and shape-memory	84.04% transmittance and shape recovery ratio up to 96.57%.	[69]
Delignified wood	Hemiacetal ester	Transparent, shape-memory, and thermal insulation	77.27% transmittance, shape recovery ratio up to 80%, and thermal conductivities as low as 0.22 W/m∙K	[70]
Delignified wood	Carboxylic ester	Shape–Color synchronous dual-response	Shape recovery ratio up to 94.62% and thermochromic at 40 °C.	[62]
Delignified wood	Carboxylic ester	Weldability and reprocessability	Recovery of 57.59% and 26.55% of the growth direction strength	[73]
Delignified wood	Imine bonds	Repairability and reprocessability	Recovery of 63.87% and 30% of the original strength.	[72]

**Table 2:** Wood substrate form, reversible bonds of DCPN, functionalities, and performance metrics of Wood powder/DCPN composites.

Wood Substrate Form	Reversible Bonds of DCPN	Functionalities	Performance Metrics	Ref.
Wood powder	Imine bonds	Repairability, reprocessibility, and closed-loop full recyclability	Recovered > 120% and 95%, >86% and >89%, and >130% and >92% tensile strength and modulus for the repaired, reprocessed, and solution recycled samples.	[75]
Wood fiber	Carboxylic ester	Reprocessibility	Recovered 67.8% flexural strength and 84.2% flexural modulus.	[78]
Wood powder	Imine bonds	Chemical degradation	Completely degraded into 5% acetic acid solution at room temperature (25 °C) within 24 h.	[76]
Wood powder	Imine bonds	Chemical degradability	Completely degraded in a diamine solution at 80 °C after 12 h.	[77]
Wood powder	Phenol carbamate	Reprocessability	Mechanical properties recovery ratio higher than 70%, even after being reprocessed three times.	[79]

## Data Availability

No new data were created or analyzed in this study.
